# Culture shapes the SNARC-like effect for visual speed

**DOI:** 10.3758/s13423-025-02793-4

**Published:** 2026-01-05

**Authors:** Mario Dalmaso, Maryam Jansarvatan, Anna Lorenzoni, Stefano Dalla Bona, Michele Vicovaro

**Affiliations:** 1 Department of Developmental and Social Psychology, University of Padova, via Venezia 8, 35131 Padova, Italy; 2 Department of General Psychology, University of Padova, via Venezia 8, 35131 Padova, Italy

**Keywords:** SNARC-like effect, Distance effect, Visual speed, Cross-cultural differences

## Abstract

Numerical and non-numerical quantities are often mapped onto horizontal space. Previous research suggests that culture-related habits, such as reading and writing direction, can influence the orientation of this mapping. While cross-cultural differences have been observed in the spatial representation of time, similar effects have not been consistently found for numbers. This discrepancy has been attributed to the fact that reading and writing involve the sequential processing of information in a specific direction but are not inherently related to numerical content. These findings support the hypothesis that the spatial representation of magnitudes depends on specific experiential associations. The present study investigated potential cross-cultural differences in the spatial representation of visual speed by comparing participants from Italy, who use a left-to-right reading and writing system, with participants from Iran, who use a right-to-left system. Participants judged whether the speed of a centrally presented random dot kinematogram was slower or faster than a reference, using left or right response keys. Results revealed opposite spatial mappings of visual speed: a left-to-right mapping for Italian participants and a right-to-left mapping for Iranian participants. These findings support the view that reading and writing direction shapes the spatial representation of magnitudes intrinsically linked to directional reading experiences, such as motion at a specific speed.

## Introduction

Humans represent numerical quantities along a spatial continuum that, in Western cultures, typically proceeds from left to right, with smaller and larger magnitudes associated with the left and right sides of space, respectively (Fias & Fischer, 2005). The Spatial-Numerical Association of Response Codes (SNARC) effect provides strong empirical evidence for this association (Dehaene et al., 1993). It refers to the fact that, in number classification tasks, responses are generally faster when smaller digits are assigned to a left-side response key and larger digits to a right-side key, than when this mapping is reversed.

Even if the actual origins of space-number associations are still debated (Eccher et al., 2025), they may originate at the biological level (e.g., Felisatti et al., 2020; Vallortigara, 2017), which could explain why they have been documented even in newborns (e.g., de Hevia et al., 2017) and non-human species (e.g., Rugani et al., 2015). In addition, and of particular relevance to this work, it has been suggested that culture-related habits may also shape space-number associations. For instance, two early studies found that in cultures where reading and writing (R&W) proceed from right to left, such as Arabic or Farsi, space-number associations tend to follow the same orientation (Dehaene et al., 1993; Shaki et al., 2009). However, despite this initial evidence, two recent studies involving Iranian participants have reported either a weakened or a regular left-to-right SNARC effect, casting doubt on the hypothesis that R&W direction directly determines the SNARC effect direction (Bulut et al., 2025; Hochman et al., 2025).

Beyond numbers, a growing body of research has also demonstrated the presence of SNARC-like effects for various non-numerical magnitudes, such as time and physical size (for a meta-analysis, see Macnamara et al., 2018). These findings support theories proposing a shared system for processing magnitudes, such as the well-known ‘A Theory of Magnitude’ (ATOM; Walsh, 2003). However, the influence of culture-related habits on the spatial representation of non-numerical magnitudes remains largely unexplored. In this regard, for instance, Vallesi et al. (2014) found a left-to-right SNARC-like effect for temporal durations in Italian participants (who have a left-to-right R&W direction) but no effect in Israeli participants (who have a right-to-left R&W direction). This result aligns well with the ‘Correlation in Experience Theory’ (CORE; Pitt & Casasanto, 2020), which suggests that R&W direction influences the spatial representation of physical dimensions inherently linked to it. Since R&W involve directional movement over time, they are expected to shape the spatial representation of time rather than numbers, a prediction also supported by initial empirical evidence (e.g., Fuhrman & Boroditsky, 2010).

In the context of SNARC-like effects, a recent study (Vicovaro et al., 2025) documented a spatial representation of visual speed. Visual speed is a particularly intriguing dimension because it is inherently tied to spatial and directional properties, providing a unique opportunity to examine how motion cues interact with the cognitive representation of magnitude along a spatial continuum. Vicovaro et al. (2025) tested Italian participants using random dot kinematograms (RDKs) as stimuli, in which a set of 100 white dots moved uniformly in different directions across a circular area. Participants categorised the speed of the moving dots as either slower or faster than a reference speed, using lateralised response keys. The results showed that slower and faster speeds were consistently associated with the left and right sides of space, respectively. This pattern emerged not only when stimuli moved left to right (Experiment 1) but also in random directions (Experiment 2) and even when motion was consistently right to left (Experiment 3), highlighting the stability of the left-to-right representation.

Visual speed is also relevant from a cross-cultural perspective. R&W involves directional motion that unfolds at a certain speed. Indeed, during R&W, individuals not only move in a specific spatial direction but also do so at a particular speed. This makes speed one of the physical dimensions inherently associated with R&W activities. According to the CORE framework, the spatial representation of visual speed may therefore be influenced by the directionality of R&W habits. Here, we explored how the spatial representation of visual speed might be influenced by right-to-left R&W direction, specifically focusing on Iranian culture. To this end, we administered an exact replication of Experiment 2 from Vicovaro et al. (2025), in which RDKs moved randomly across a 360° spectrum. This allowed us to clearly isolate the influence of cultural factors on the spatial representation of visual speed by removing consistent directional cues from the stimuli, which were instead present in the other experiments of Vicovaro et al. (2025).

## Method

### Participants

We tested 30 participants (mean age = 28.9 years, *SE* = 0.83; 12 males; six left-handed). Sample size (identical to Vicovaro et al. 2025, Experiment 2) was determined following Brysbaert and Stevens (2018), who recommend at least 1,600 data points per condition in reaction time (RT) studies to ensure statistical power. Our 2 × 2 within-participant design (response side: left or right; target speed: slower or faster) with 60 trials per condition (see the *Procedure* section), required at least 27 participants. Based on the data of Vicovaro et al.’s (2025) Experiment 2, we also conducted a power estimation based on Monte Carlo simulations, which indicated that our design and analyses had approximately .88 power to detect a SNARC-like effect of the same magnitude. The R scripts containing detailed analyses and results are available on the Open Science Framework (OSF; please see *Availability of data and materials*).

All participants were Iranian students enrolled in different courses at the University of Padova. Handedness was further assessed through self-report and the Edinburgh Handedness Inventory (EHI; Veale, 2014). The mean EHI score was 57.9 (*SE* = 14; range = −100 to +100). Seven participants had an EHI score ≤ ˗50 (indicating a left-hand preference), and the remaining had an EHI score ≥75 (indicating a right-hand preference).

Other personal information was collected through a written questionnaire (see the *Procedure* section). Most participants were students from various academic fields, including psychology, engineering, economics, and management, with one postdoctoral researcher in mathematics. On a scale from 0 to 7, English proficiency (*M* = 5.70, *SE* = 0.18) and daily use of English (*M* = 5.83, *SE* = 0.16) were rated as relatively high, and musical practice was rated as minimal (*M* = 0.43, *SE* = 0.20). Time spent in Western countries ranged from 0.5 to 6 years (*M* = 2.08, *SE* = 0.13).

The study was approved by the Ethics Committee for Psychological Research at the University of Padova (approval number: 4480) and conducted in accordance with the Declaration of Helsinki. Written informed consent (written, from right to left, in Farsi) was obtained from all participants before participating in the experiment.

### Apparatus

We used the same apparatus adopted by Vicovaro et al. (2025, Experiment 2). The experimental script was developed using PsychoPy3 (Peirce et al., 2022) and displayed on an LCD screen (35.54 cm × 20 cm, 1,366 × 768 pixels, 60 Hz). Participants were seated approximately 50 cm from the screen, which featured a grey background. Manual responses were collected using a standard keyboard positioned centrally in front of the screen.

**Fig. 1:**
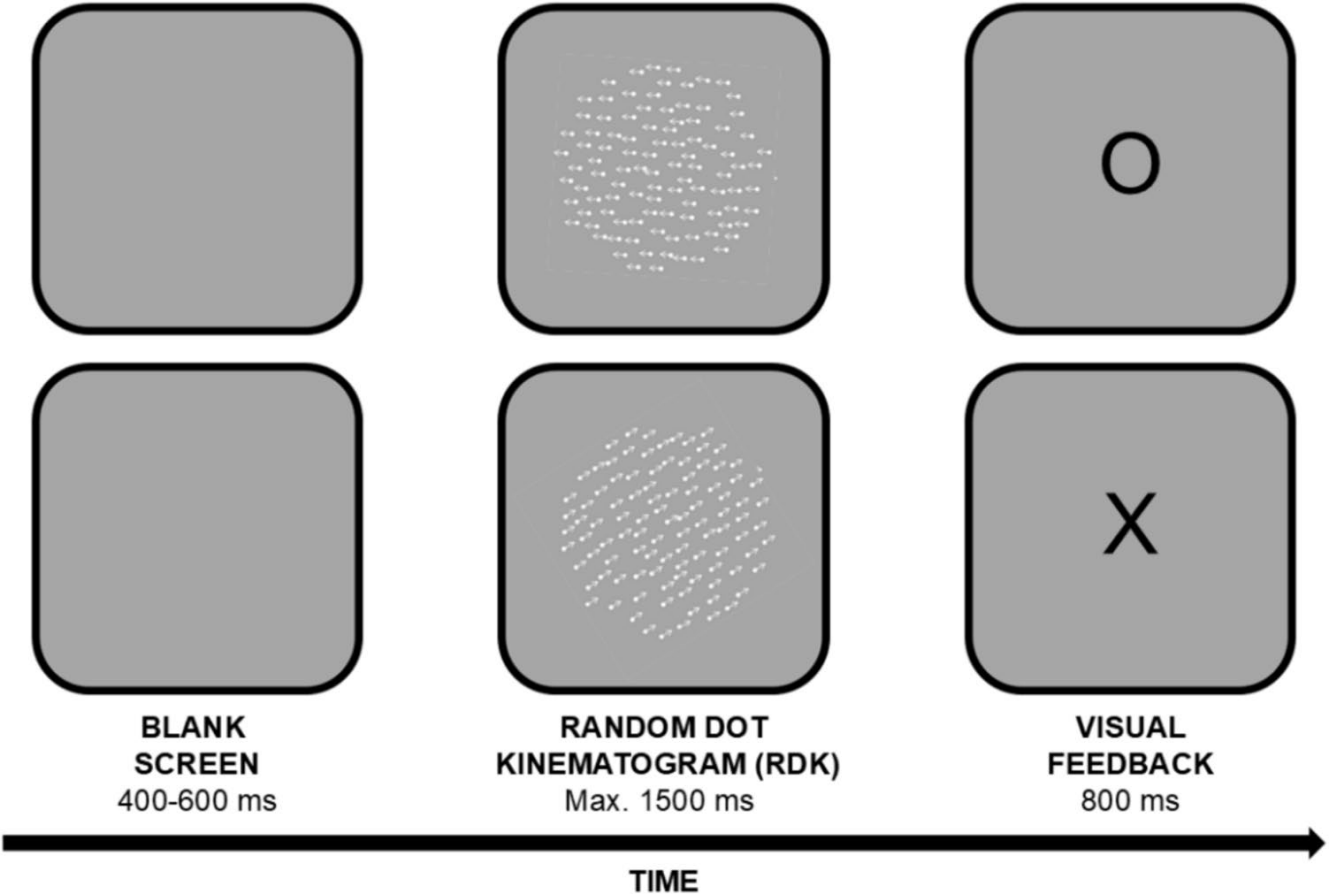
Trial illustration and stimuli (not to scale). Arrows in the random dot kinematogram (RDK) show motion direction (right to left in the upper panel, bottom-left to top-right in the lower panel) and are for illustration only. Feedback ‘O’ indicates a correct response, while ‘X’ indicates an incorrect one

The stimuli were random dot kinematograms (RDKs; see Fig. 1) created using the Dots tool in the PsychoPy3 Builder. Each RDK consisted of 100 white dots moving in the same direction and with the same speed within a 10-cm circular area. Dots had a size of 5 pixels, a lifespan of 60 frames (1 s), and were replaced upon reaching the boundary or completing their lifespan, ensuring a constant dot count per frame. The maximum duration of RDKs was 1.5 s. In each trial, the dots in the RDK moved in a randomly selected direction between 0° and 360°, with all dots sharing the same direction and speed. A reference speed of 8 cm/s was compared to 20 target speeds: 10 slower (5.5 to 7.75 cm/s) and 10 faster (8.25 to 10.5 cm/s), each varying in 0.25 cm/s increments.

### Procedure

The procedure was identical to that of Vicovaro et al. (2025, Experiment 2), with the exception that participants were welcomed and instructed by an Iranian experimenter speaking Farsi, and all on-screen materials were presented in Farsi, written from right to left. Participants were required to determine whether the speed of moving dots was faster or slower than the reference speed. They used the ‘A’ and ‘L’ keys for their responses. The key assignment for ‘faster’ and ‘slower’ was counterbalanced across participants. The ‘A’ key was covered with a red sticker and the ‘L’ key with a blue sticker. Participants were instructed how to respond by referring to the key colours rather than the letters to avoid any potential interference from direct exposure to the Latin alphabet. The instructions emphasised the need for both speed and accuracy in their responses.

At the beginning of the procedure, participants observed the reference speed twice, each instance separated by a 500-ms blank screen. The randomly chosen direction of the dots was kept consistent across the two presentations of the target speed. Participants then completed a practice block of 20 trials, where each trial began with a blank screen (random duration from 400 to 600 ms), followed by the presentation of a target speed selected at random. After each trial, feedback was shown for 800 ms: ‘O’ for correct responses, ‘X’ for incorrect ones, and ‘TOO SLOW’ (written, from right to left, in Farsi) if no response was made before the time-out limit of 1.5 s. Practice trial responses were excluded from the analysis. The experimental block mirrored the practice block. It consisted of 120 trials (20 target speeds repeated six times) presented randomly. Participants were shown the reference speed twice after every 30 trials. Following the first experimental block, the response-key mapping was reversed, and participants completed another practice block and experimental block. Across both experimental blocks, a total of 240 trials were conducted, split equally between the two.

At the end of the experiment, participants completed a brief questionnaire providing their age, sex, handedness (i.e., left, right, both), and field of study or work. They rated their English proficiency, daily use of English, and music practice (if applicable) on a scale from 0 to 7. They also indicated how many years they had lived in a Western country.

## Results

Incorrect responses (14.74%) were excluded and analysed separately.^1^ All participants performed above chance, with a maximum error rate of 24.17%. Missed responses (0.65%) were excluded due to their low frequency, and outliers (i.e., correctly responded trials with a RT smaller or higher than three standard deviations from the participant’s mean) were also removed (1.25%).

Raw RTs for correctly responded trials were analysed using generalised linear mixed-effects models (R package *lme4*; Bates et al., 2015) with an identity link function for a Gamma-distributed response variable (Lo & Andrews, 2015). The fixed effects were response side (left or right key), target speed (slower or faster than the reference), and their interaction. To align with the assumption that explicit magnitude classification tasks follow a step function relationship between target magnitude and RTs (Gevers et al., 2006), speed was treated as a binary variable.^2^ Random effects varied across models and included by-participant and by-item intercepts, along with by-participant slopes for speed and response side and by-item slope for response side. Here, ‘items’ referred to the 20 distinct target speeds. Likelihood ratio tests were used to compare models with increasing random effect complexity.

**Fig. 2:**
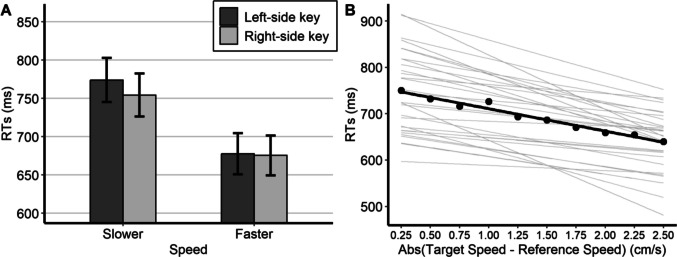
Panel A: Model-based estimates of mean reaction times (RTs; bars) from Iranian participants, displayed by relative speed and response side, with error bars indicating estimated SEM. Panel B: Illustration of the distance effect, showing the estimated fixed effect (thick line), the estimated random effects (thin lines), and the observed mean RTs (dots)

The best-fitting model included, as random effects, the by-participant and by-item intercepts and the by-participant slopes for speed and response side. A Type 3 Wald chi-square test was then applied to the model (R package *car*; Fox & Weisberg, 2019). The main effect of response side was not significant, χ^2^(1) = 1.68, *p* = 0.195, but the main effect of target speed was significant, χ^2^(1) = 10.07, *p* = 0.0015, indicating faster responses for faster target speeds (*M* = 677 ms, *SE* = 26.1) compared to slower target speeds (*M* = 764 ms, *SE* = 28.1). Importantly, the interaction coefficient was significant, χ^2^(1) = 6.50, *p* = 0.011, confirming the presence of a SNARC-like effect.^3^ Further comparisons with the *emmeans* function (Lenth, 2023) revealed that for faster target speeds there was no significant difference (*p* = 0.803) between the left (*M* = 678 ms, *SE* = 26.9) and right (*M* = 675 ms, *SE* = 26.1) response key. However, for slower target speeds, a significant difference (*p* = 0.035) emerged between the left (*M* = 774 ms, *SE* = 28.9) and the right (*M* = 754 ms, *SE* = 28.0) response key (see also Fig. 2, panel A). This pattern of results supports the hypothesis of a right-to-left representation of visual speed among Iranian participants, in contrast to the pattern observed among Italian participants.

We also assessed the distance effect, analysing whether absolute RTs decreased as the absolute difference between target and reference speeds increased (e.g., Moyer & Landauer, 1967). A generalised linear mixed-effects model (identity link function and Gamma-distributed response variable), with distance as a fixed effect and by-participant random intercept and slope for distance, confirmed the effect, *b* = −0.048, *SE* = 0.007, χ^2^(1) = 49.81, *p* < 0.001 (see also Fig. 2, panel B).

### Comparisons between the current study and Experiment 2 of Vicovaro et al. (2025)

For completeness, we directly compared the current data with those of Experiment 2 of Vicovaro et al. (2025). As for RTs of correctly responded trials, the best-fitting model included response side, target speed, and group (Iranians or Italians), and their interactions as fixed effects, whereas as random effects it included the by-participant and by-item intercepts and the by-participant slope for response side and target speed. Briefly, while the main effect of group was non-significant, χ^2^(1) = 0.03, *p* = 0.866, the response side × target speed × group interaction was significant, χ^2^(1) = 18.01, *p* < 0.001, in line with the hypothesis that the spatial representation of speed was different within the two groups.^4^ As for accuracy, the best model had the same structure as that used for RTs. The only significant result was the main effect of the group, χ^2^(1) = 9.11, *p* = 0.003, indicating a slightly higher percentage of errors among Italians (18.22%) than Iranians (14.74%). Finally, the distance effect, analysed with a model including distance, group, and their interaction as fixed effects, and the by-participant intercept as random effect, revealed no significant interaction between distance and group, *b* = 0.011, *SE* = 0.009, χ^2^(1) = 1.53, *p* = 0.217, suggesting that the distance effect had similar magnitude across Iranian and Italian participants.

## Discussion

We investigated how cultural factors influence the SNARC-like effect for visual speed by testing Iranian participants in a replication of Experiment 2 from Vicovaro et al. (2025), which originally involved Italian participants. The goal was to determine whether the spatial representation of visual speed in Iranians aligns with the right-to-left R&W direction of Farsi. The results supported this prediction: a SNARC-like effect emerged and, more importantly, the spatial representation of visual speed followed a right-to-left orientation, contrasting with the left-to-right representation observed in Italians.

The right-to-left representation of visual speed observed in Iranians is particularly noteworthy in light of recent findings showing a left-to-right SNARC effect in the same population (Bulut et al., 2025; Hochman et al., 2025). The contrast between the spatial representation of speed and numbers lends indirect support to CORE theory (Pitt & Casasanto, 2020), which predicts that R&W direction influences space-magnitude associations only for magnitudes inherently linked to R&W. Given that R&W entails motion in a specific direction at a certain speed, the influence of R&W direction on visual speed seems to align with the predictions of CORE theory.

One could argue that R&W direction is not the only cultural habit that might explain the observed differences between Iranian and Italian participants (see also Bulut et al., 2025). Other culturally influenced factors, such as finger-counting direction and number-reading direction, may also contribute to space-magnitude associations. However, given the characteristics of these habits in Iranian participants, it is hard to make clear predictions regarding the expected direction of these associations. Indeed, Iranians typically count with palms facing upward, starting from the small finger of the right hand, which follows a left-to-right sequence (Lindemann et al., 2011). Meanwhile, multi-digit numbers are generally read and written from left to right (e.g., 163), whereas single digits (1–10) are often represented from right to left (Bulut et al., 2025). Among these alternatives, R&W direction stands out as the most plausible explanation for the right-to-left representation of visual speed observed in Iranian participants.

To gather more information on directional preferences in Iranian participants, we also decided to administer the Cultural Directional Preferences Questionnaire (CDPQ), developed by Bulut et al. (2025). This tool was created to capture habitual spatial biases in various conceptual domains, such as object placement, imagined movement, and depiction of quantity. The CDPQ provides an index of individual preferences for representing concepts along the horizontal axis. Scores range from −7 to +7, with negative values indicating a preference for left-to-right representations and positive values indicating a preference for right-to-left representations. It was administered by Bulut et al. (2025) to large samples of Germans living in Germany and Iranians living in Iran. We translated the English version of the CDPQ into Farsi and administered it to the Iranian participants of the current experiment via Qualtrics. Because the questionnaire was published after our experimental data collection had been completed, it was administered separately after the main experiment. Nonetheless, we aimed to complement the behavioural findings with an explicit measure of directional spatial preferences.

In the original study by Bulut et al. (2025), German participants showed a robust left-to-right preference (*M* = − 3.65), while Iranian participants showed no clear preference (*M* = 0.07). In our sample, we observed a similar pattern: the mean score among Iranian participants was slightly negative (*M* = − 0.8, *SE* = 0.414), suggesting a weak tendency toward left-to-right representation. However, this value did not significantly differ from zero (*p* = .063; data and analyses are available on the OSF, see *Availability of data and materials*), indicating no systematic spatial bias at the group level. This pattern, consistent with the findings of Bulut et al. (2025), may reflect a tension between biologically rooted left-to-right biases and culturally acquired right-to-left habits, especially in populations exposed to mixed cultural influences. Notably, all of our Iranian participants were living in a Western country at the time of testing, yet their responses were similar to those of the Iran-based sample from Bulut et al. (2025). This suggests that individual spatial directional preferences may remain relatively stable despite immersion in a different cultural context, at least over the short to medium term.

Importantly, a right-to-left SNARC-like effect for visual speed emerged despite the absence of a strong directional bias at the explicit level. This dissociation reinforces the idea that implicit spatial-magnitude mappings, such as those revealed in speed classification tasks, may operate independently of consciously reported preferences. Moreover, the similarity in explicit directional preferences between our Iranian participants and those tested by Bulut et al. (2025) suggests that the observed dissociation (i.e., the presence of a clear right-to-left SNARC-like effect for visual speed in our study, but not of a clear right-to-left SNARC effect for numbers in Bulut et al., 2025) cannot be attributed to systematic differences in explicit preferences. Instead, it more likely reflects that R&W direction selectively influences the spatial representation of dimensions intrinsically linked to literacy-related activities, such as time and speed.

Beyond the general explanation provided by the CORE framework, it is also worth considering a complementary hypothesis regarding the observed effect of R&W direction on the SNARC-like effect for visual speed. A central characteristic of our task was that participants were required, at the beginning of the experiment, to encode the reference speed and retain it for the entire duration of the experiment. Consequently, this continuous need to keep the reference active likely engaged working memory. Intriguingly, previous studies have shown that information maintained in working memory can be spatially organised according to R&W direction, an effect known as the Spatial-Ordinal Association of Response Codes (SPoARC; van Dijck & Fias, 2011; Guida et al., 2018; see also Rasoulzadeh et al., 2024). Therefore, it is plausible that, also in the present context, working memory may have played a role in shaping the observed SNARC-like effect reported here.

The following discussion focuses on specific results from the comparison between our Iranian sample and the Italian sample tested in Vicovaro et al.’s (2025) Experiment 2. First, in both groups, RTs were shorter for fast speeds than for slow speeds. As suggested by Vicovaro et al. (2025), this may be because participants responded only after the dots had travelled a minimum distance, which fast-moving dots reached sooner than slow-moving ones (see also Tynan & Sekuler, 1982). However, it is worth noting that a similar advantage for faster over slower stimuli has also been reported in the auditory domain (Mariconda et al., 2024), likely suggesting a generalised processing benefit for high-speed information. Second, an asymmetry emerged in the response patterns across groups. Among Iranians, a significant difference between right and left response keys was found only for slower speeds, whereas for Italians, the opposite pattern occurred, with a significant difference emerging only for faster speeds. This asymmetry is likely related to the predominance of right-handed participants in both groups. Right-hand dominance may have amplified RT differences for stimuli preferentially represented on the right (slow speeds for Iranians, fast speeds for Italians) while reducing differences for stimuli represented on the left. Such imbalances in response-side effects across small and large magnitudes are not uncommon in SNARC-like research (e.g., Chang & Cho, 2015; Dalmaso et al., 2019, 2023a, b).

In conclusion, our findings indicate that visual speed is mapped onto space according to the native R&W direction. By comparing Iranians with the Italian participants from Vicovaro et al. (2025), this study contributes to cross-cultural research on SNARC-like effects (e.g., Shaki et al., 2012; Vallesi et al., 2014), a field that remains relatively underexplored. These insights deepen our understanding of how culture and cognition intertwine, shedding light on the interplay between universal cognitive processes and the culturally shaped ways in which humans perceive and structure the world.

## Data Availability

Data and materials are available on the Open Science Framework (OSF): 10.17605/OSF.IO/PGSHD. The study was not pre-registered. Data from Vicovaro et al. (2025) are also available on the OSF: 10.17605/OSF.IO/WNM9S

## Footnotes

1 Errors were analysed with generalised linear mixed-effects models (R package lme4; Bates et al., 2015), using a logit link function for a binomially distributed response variable. The best-fitting model included response side (left or right key), target speed (slower or faster than the reference), and their interaction as fixed effects, while as random effects it included the by-participant and by-item intercepts, the by-participant slopes for speed and response side, and the by-item slope for response side. No significant results emerged (*p*s ≥ 0.136).

2 The validity of this assumption was supported by visual inspection of the distribution of individual delta RTs (i.e., the by-participant difference between mean RTs for the right and left keys) across speed levels, which closely resembled a step function (see Online Supplementary Material Fig. A on the OSF).

3 A Monte Carlo simulation indicated that, under the null hypothesis of no effect of response keys on RTs, the probability of obtaining a statistically significant interaction in the same direction as that observed was .045, suggesting adequate control of the Type I error rate.

4 For Italian participants, the interaction between target speed and response side was statistically significant (χ^2^(1) = 11.58, *p* < 0.001), with the direction of the effect opposite to that observed for Iranian participants. Specifically, for faster target speeds, a significant difference (*p* < 0.001) emerged between the left (*M* = 679 ms, *SE* = 25.4) and the right (*M* = 661 ms, *SE* = 25.4) response key. For the slower target speeds, there was no significant difference (*p* = 0.200) between the left (*M* = 759 ms, *SE* = 25.3) and right (*M* = 766 ms, *SE* = 25.5) response keys.

## Code availability

Experiment and analysis codes are available on the OSF: 10.17605/OSF.IO/PGSHD

## References

[CR1] Bates, D., Mächler, M., Bolker, B., & Walker, S. (2015). Fitting linear mixed-effects models using lme4. *Journal of Statistical Software,**67*, 1–48. 10.18637/jss.v067.i01

[CR2] Brysbaert, M., & Stevens, M. (2018). Power analysis and effect size in mixed effects models: A tutorial. *Journal of Cognition,**1*(1), 9. 10.5334/joc.10 31517183 10.5334/joc.10 PMC6646942

[CR3] Bulut, M., Roth, L., Bahreini, N., Cipora, K., Reips, U. D., & Nuerk, H. C. (2025). One direction? Cultural aspects of the mental number line beyond reading direction. *Psychological Research,**89*, 37. 10.1007/s00426-024-02038-4 10.1007/s00426-024-02038-4 PMC11663824 39710792

[CR4] Chang, S., & Cho, Y. S. (2015). Polarity correspondence effect between loudness and lateralized response set. *Frontiers in Psychology,**6*, Article 683. 26052305 10.3389/fpsyg.2015.00683 PMC4440908

[CR5] Dalmaso, M., Pileggi, S., & Vicovaro, M. (2023a). Face age is mapped into three-dimensional space. *Cognitive Science,**47*(11), Article e13374. 10.1111/cogs.13374 37950541 10.1111/cogs.13374

[CR6] Dalmaso, M., Schnapper, Y., & Vicovaro, M. (2023b). When time stands upright: STEARC effects along the vertical axis. *Psychological Research,**87*(3), 894–918. 10.1007/s00426-022-01693-9 35718808 10.1007/s00426-022-01693-9 PMC10017642

[CR7] Dalmaso, M., & Vicovaro, M. (2019). Evidence of SQUARC and distance effects in a weight comparison task. *Cognitive Processing,**20*, 163–173. 10.1007/s10339-019-00905-2 30721375 10.1007/s10339-019-00905-2

[CR8] Dehaene, S., Bossini, S., & Giraux, P. (1993). The mental representation of parity and number magnitude. *Journal of Experimental Psychology: General,**122*(3), 371–396. 10.1037/0096-3445.122.3.371

[CR9] de Hevia, M. D., Veggiotti, L., Streri, A., & Bonn, C. D. (2017). At birth, humans associate “few” with left and “many” with right. *Current Biology,**27*(24), 3879–3884, e2. 10.1016/j.cub.2017.11.024 29225024 10.1016/j.cub.2017.11.024

[CR10] Eccher, E., Josserand, M., Caparos, S., Boissin, E., Buiatti, M., Piazza, M., & Vallortigara, G. (2025). A left-to-right bias in number-space mapping across ages and cultures. *Nature Communications,**16*, 495. 10.1038/s41467-024-55685-x 39794321 10.1038/s41467-024-55685-x PMC11724025

[CR11] Felisatti, A., Laubrock, J., Shaki, S., & Fischer, M. H. (2020). A biological foundation for spatial-numerical associations: The brain’s asymmetric frequency tuning. *Annals of the New York Academy of Sciences,**1477*(1), 44–53. 10.1111/nyas.14418 32645221 10.1111/nyas.14418

[CR12] Fias, W., & Fischer, M. H. (2005). Spatial representation of numbers. *The handbook of mathematical cognition* (pp. 43–54). Psychology Press.

[CR13] Fox, J., & Weisberg, S. (2019). *An R companion to applied regression* (3rd ed). Sage.

[CR14] Fuhrman, O., & Boroditsky, L. (2010). Cross-cultural differences in mental representations of time: Evidence from an implicit nonlinguistic task. *Cognitive Science,**34*, 1430–1451. 10.1111/j.1551-6709.2010.01105.x 21564254 10.1111/j.1551-6709.2010.01105.x

[CR15] Gevers, W., Verguts, T., Reynvoet, B., Caessens, B., & Fias, W. (2006). Numbers and space: A computational model of the SNARC effect. *Journal of Experimental Psychology. Human Perception and Performance,**32*, 32–44. 10.1037/0096-1523.32.1.32 16478324 10.1037/0096-1523.32.1.32

[CR16] Guida, A., Megreya, A. M., Lavielle-Guida, M., Noël, Y., Mathy, F., van Dijck, J. P., & Abrahamse, E. (2018). Spatialization in working memory is related to literacy and reading direction: Culture “literarily” directs our thoughts. *Cognition,**175*, 96–100. 10.1016/j.cognition.2018.02.013 29486378 10.1016/j.cognition.2018.02.013

[CR17] Hochman, S., Havedanloo, R., Heysieattalab, S., & Soltanlou, M. (2025). How does language modulate the association between number and space? A registered report of a cross-cultural study of the spatial-numerical association of response codes effect. *Journal of Experimental Psychology. General,**154*(2), 305–324. 10.1037/xge0001653 39621408 10.1037/xge0001653

[CR18] Lenth, R. V. (2023). *emmeans: Estimated marginal means, aka least-squares means* (Version 1.8.4-1) [R package]. Comprehensive R Archive Network (CRAN). https://CRAN.R-project.org/package=emmeans

[CR19] Lindemann, O., Alipour, A., & Fischer, M. H. (2011). Finger counting habits in Middle Eastern and Western individuals: An online survey. *Journal of Cross-Cultural Psychology,**42*(4), 566–578. 10.1177/0022022111406254

[CR20] Lo, S., & Andrews, S. (2015). To transform or not to transform: Using generalized linear mixed models to analyse reaction time data. *Frontiers in Psychology,**6*, Article 1171. 10.3389/fpsyg.2015.01171 26300841 10.3389/fpsyg.2015.01171 PMC4528092

[CR21] Macnamara, A., Keage, H. A., & Loetscher, T. (2018). Mapping of non-numerical domains on space: A systematic review and meta-analysis. *Experimental Brain Research,**236*(2), 335–346. 10.1007/s00221-017-5154-6 29279982 10.1007/s00221-017-5154-6

[CR22] Mariconda, A., Murgia, M., De Tommaso, M., Mingolo, S., Agostini, T., & Prpic, V. (2024). Temporal speed prevails on interval duration in the SNARC-like effect for tempo. *Attention, Perception, & Psychophysics,**86*(1), 263–272. 10.3758/s13414-023-02816-z 10.3758/s13414-023-02816-z PMC10770242 37985595

[CR23] Moyer, R. S., & Landauer, T. K. (1967). Time required for judgements of numerical inequality. *Nature,**215*(5109), 1519–1520. 10.1038/2151519a0 6052760 10.1038/2151519a0

[CR24] Peirce, J. W., Hirst, R. J., & MacAskill, M. R. (2022). *Building Experiments in PsychoPy* (2nd ed). Sage.

[CR25] Pitt, B., & Casasanto, D. (2020). The correlations in experience principle: How culture shapes concepts of time and number. *Journal of Experimental Psychology: General,**149*(6), 1048–1070. 10.1037/xge0000696 31633369 10.1037/xge0000696

[CR26] Rasoulzadeh, V., van Dijck, J. P., Khosrowabadi, R., & Fias, W. (2024). Reading direction interacts with spatial processes of temporal order verbal working memory: Evidence from Iranian right-to-left readers. *Language, Cognition and Neuroscience,**39*(8), 1072–1089. 10.1080/23273798.2024.2370046

[CR27] Rugani, R., Vallortigara, G., Priftis, K., & Regolin, L. (2015). Animal cognition. Number-space mapping in the newborn chick resembles humans’ mental number line. *Science,**347*(6221), 534–536. 10.1126/science.aaa1379 25635096 10.1126/science.aaa1379

[CR28] Shaki, S., Fischer, M. H., & Petrusic, W. M. (2009). Reading habits for both words and numbers contribute to the SNARC effect. *Psychonomic Bulletin & Review,**16*(2), 328–331. 10.3758/PBR.16.2.328 19293102 10.3758/PBR.16.2.328

[CR29] Shaki, S., Petrusic, W. M., & Leth-Steensen, C. (2012). SNARC effects with numerical and non-numerical symbolic comparative judgments: Instructional and cultural dependencies. *Journal of Experimental Psychology. Human Perception and Performance,**38*(2), 515–530. 10.1037/a0026729 22288694 10.1037/a0026729

[CR30] Tynan, P. D., & Sekuler, R. (1982). Motion processing in peripheral vision: Reaction time and perceived velocity. *Vision Research,**22*(1), 61–68. 10.1016/00426989(82)90167-5 7101752 10.1016/0042-6989(82)90167-5

[CR31] Vallesi, A., Weisblatt, Y., Semenza, C., & Shaki, S. (2014). Cultural modulations of space–time compatibility effects. *Psychonomic Bulletin & Review,**21*, 666–669. 10.3758/s13423-013-0540-y 24163172 10.3758/s13423-013-0540-y

[CR32] Vallortigara, G. (2017). Comparative cognition of number and space: The case of geometry and of the mental number line. *Philosophical Transactions of the Royal Society of London. Series B, Biological Sciences,**373*(1740), Article 20170120. 10.1098/rstb.2017.0120 29292353 10.1098/rstb.2017.0120 PMC5784052

[CR33] van Dijck, J. P., & Fias, W. (2011). A working memory account for spatial–numerical associations. *Cognition,**119*(1), 114–119. 10.1016/j.cognition.2010.12.013 21262509 10.1016/j.cognition.2010.12.013

[CR34] Veale, J. F. (2014). Edinburgh handedness inventory - Short form: A revised version based on confirmatory factor analysis. *Laterality,**19*(2), 164–177. 10.1080/1357650X.2013.783045 23659650 10.1080/1357650X.2013.783045

[CR35] Vicovaro, M., Boscariol, R., & Dalmaso, M. (2025). A SNARC-like effect for visual speed. *Attention, Perception, & Psychophysics,**87*, 1042–1059. 10.3758/s13414-025-03012-x 10.3758/s13414-025-03012-x PMC11965142 39881113

[CR36] Walsh, V. (2003). A theory of magnitude: Common cortical metrics of time, space and quantity. *Trends in Cognitive Sciences,**7*(11), 483–488. 10.1016/j.tics.2003.09.002 14585444 10.1016/j.tics.2003.09.002

